# Differential item functioning between English, German, and Spanish PROMIS® physical function ceiling items

**DOI:** 10.1007/s11136-024-03866-y

**Published:** 2024-12-16

**Authors:** Constantin Yves Plessen, Felix Fischer, Claudia Hartmann, Gregor Liegl, Ben Schalet, Aaron J. Kaat, Rodrigo Pesantez, Alexander Joeris, Marilyn Heng, Matthias Rose, the AOBERT Consortium, Mark Vrahas, Stephen E. Gwilym, Marcel Orth, Benedikt J. Braun, Peter Augat, Nan E. Rothrock, Livio Di Mascio, Tim Pohlemann, Prakash Jayakumar, Stefan Dobele, Martin Jaegar

**Affiliations:** 1 Center for Patient-Centered Outcomes Research, Medizinische Klinik mit Schwerpunkt für Psychosomatik, Charité – Universitätsmedizin Berlin, corporate member of Freie Universität Berlin and Humboldt Universität zu Berlin, Charitéplatz 1, 10097 Berlin, Germany; 2 German Center for Mental Health (DZPG), Berlin, Germany; 3 Institute for Biomechanics, BG Unfallklinik, Murnau, Germany; 4 Paracelsus Medical University, Salzburg, Austria; 5 Department of Trauma, Hand and Reconstructive Surgery, Saarland University, Homburg, Germany; 6 Department of Medical Social Science, Northwestern University Feinberg School of Medicine, Chicago IL, USA; 7 University Hospital Tuebingen; on behalf of the Eberhard-Karls-University Tuebingen, Faculty of Medicine, BG Hospital, Tuebingen, Germany; 8 AO Innovation Translation Center, AO Foundation, Davos, Switzerland; 9 Department of Orthopaedics, University of Miami Miller School of Medicine, Miami FL, USA; 10 AO Trauma, AO Foundation, Davos, Switzerland; 11 Department of Epidemiology and Data Science, Amsterdam University Medical Centers, Amsterdam, The Netherlands

**Keywords:** PROMIS, Differential item functioning, Sensitivity analysis, Physical function

## Abstract

**Purpose:**

We investigated the validity of the German and Spanish translations of 35 new high functioning items added to the Patient Reported Outcomes Measurement Information System (PROMIS®) Physical Function item bank 2.0. We assessed differential item functioning (DIF) between three general population samples from Argentina, Germany, and the United States.

**Methods:**

PROMIS Physical Function data was collected in online panels from 3601 individuals (mean age, 41.6 years old; range, 18–88 years; 53.7% female). Of these, 1001 participants completed the Spanish version, 1000 completed the German version, and 1600 completed the English version. DIF was assessed by a multiverse analysis that systematically varied analytic choices across the entire range of plausible options within the logistic ordinal regression framework.

**Results:**

Translated items generally met the assumptions of unidimensionality, monotonicity, and local independence. The 272 different analyses suggest consistent DIF between languages in four items. Test characteristic curves suggested that the magnitude and impact of DIF on the test scores were negligible for all items at the test level. After correcting for potential DIF, we observed greater scoring for physical functioning in Argentina compared to the US, Cohen’s *d* = 0.25, [0.17, 0.33], and Argentina compared to Germany, Cohen’s *d* = 0.23, [0.15, 0.32].

**Conclusions:**

Our findings support the universal applicability of PROMIS Physical Function items across general populations in Argentina, Germany, and the U.S. The sensitivity analyses indicate that the identification of DIF items was robust for different data analytic decisions. Multiverse analysis is a promising approach to address lack of clear cutoffs in DIF identification.

**Supplementary Information:**

The online version contains supplementary material available at 10.1007/s11136-024-03866-y.

## Plain English summary

We wanted to find out whether new questions added to a well-established health questionnaire assessing the ability to perform valued life activities worked similarly in German and Spanish translations compared to the original English version. We tested this by asking over 3600 people from Argentina, Germany, and the U.S. to fill out the questionnaire. We then checked if any questions were answered differently compared to the underlying construct being assessed in each country. We found differences in how a few questions were perceived in Germany and Argentina compared to the U.S., but these differences were very small and did not substantially impact the overall scores. After accounting for these differences, participants in Argentina and Germany scored higher than those in the U.S. regarding physical abilities. Overall, our study shows that these questions are useful and can be used in different countries without any major differences.

## Introduction

Self-reported physical function (PF) is an important outcome measure in patients recovering from fractures, undergoing physical rehabilitation, and gauging health status and mobility in those living with medical conditions [1, 9, 26]. Historically, the predominant limitation in traditional PF metrics has been the pronounced floor and ceiling effects, which require large sample sizes and correspondingly elevated study costs [2, 6, 16]. The PROMIS Physical Function item bank v1.2, with its 121 items, was an improvement over its predecessors, offering a broader measurement range [14]. However, certain ceiling effects persisted [2, 16], making it difficult to differentiate those with high levels of functioning. To address this limitation, the updated PROMIS Physical Function item bank v2.0 aimed to increase the measurement range, particularly at the higher end of physical ability [16]. This was achieved by introducing 35 new items.

These newly introduced items were developed in English and were not yet available in the German or Spanish versions of the PROMIS PF item banks. We translated and culturally adapted the 35 new ceiling extension items (v2.0) into both German and Universal Spanish.

Ensuring the translated items of the PROMIS Physical Function item bank version 2.0 have similar measurement properties across different languages is crucial for their validity and reliability. To achieve this, we need to assess their psychometric properties with a focus on measurement invariance among English-, German-, and Spanish-speaking populations. Measurement non-invariance, also known as differential item functioning (DIF), occurs when individuals from different countries, who have the same underlying physical function level, respond differently to a particular item. Addressing DIF is essential as it ensures cultural fairness and accuracy in measurement, promoting valid comparisons across diverse populations [24].

Ensuring the translated items of the PROMIS Physical Function item bank version 2.0 have similar measurement properties across different languages is crucial for their validity and reliability. To achieve this, we need to assess their psychometric properties with a focus on measurement invariance among English-, German-, and Spanish-speaking populations. Measurement non-invariance, also known as differential item functioning (DIF), occurs when individuals from different countries, who have the same underlying physical function level, respond differently to a particular item. Addressing DIF is essential as it ensures cultural fairness and accuracy in measurement, promoting valid comparisons across diverse populations [24].

There is no consensus on the best method to assess DIF [24], leading to a variety of statistical frameworks and methods available [12, 18]. PROMIS often relies on assessing DIF within a logistic regression framework [13]. However, even within this framework, there is no agreement on the specific cutoffs to reliably identify items with meaningful DIF. Best practices recommend distinguishing statistically significant DIF from those with practical or impactful effects, necessitating the evaluation of multiple DIF impact measures [11].

To address this knowledge gap, we assessed DIF among English, Spanish, and German items using a comprehensive psychometric sensitivity analysis within the logistic ordinal regression framework. This analysis included a wide range of defensible model specifications. We explored the implications of several factors on the outcomes, including: (1) conducting DIF analyses collectively for all countries or pairwise, (2) ignoring or adjusting for age differences among samples, (3) estimating sample-specific item parameters or using PROMIS item parameters, and (4) using different criteria for identifying items demonstrating DIF.

Our study aimed to evaluate whether the new ceiling item translations of the PROMIS Physical Function item bank version 2.0 into German and Spanish exhibit similar measurement properties as the original English version. Furthermore, we conducted extensive sensitivity analyses to ensure the robustness of our findings under a wide range of model specifications.

## Methods

### PROMIS physical function item bank and its translation

Physical Function (PF) is the ability to perform activities requiring physical actions, which range from basic self-care to more complex tasks needing various skills, often within social contexts [8]. The PROMIS Physical Function item bank is an Item Response Theory (IRT)-based calibration of a graded response model, which allows for any subset of these items to measure an individual's PF on a standardized T-score scale, representing an average of 50 and a standard deviation of 10 in the general population [14, 15]. The PROMIS PF version 1.2 itembank consists of 121 items that evaluate the ability to perform tasks using the upper extremities (such as hand dexterity), lower extremities (such as walking and level of mobility), and central body areas (neck and back), alongside the ability to undertake instrumental daily living activities, such as running errands. For the development of PROMIS PF 2.0 item bank, 35 additional items were added to extend the measurement range, in particular for individuals with good physical functioning.

These 35 items were translated into German and Spanish following the PROMIS Standards [13]. The process included creating a glossary for accurate term translation, forward and backward translations by native speakers, and cultural adaptations for regional differences. The German and Spanish versions were refined through cognitive debriefing with participants from Germany and Argentina, using feedback to ensure clarity and cultural relevance. At least 5 cognitive interviews are conducted to ensure the quality and appropriateness of the translations. The project was overseen by the PROMIS Translation Director, who ensured consistency and finalized the translations with certification, emphasizing the rigorous approach to maintaining the integrity and universality of the translations.

### Data collection

Data from the general population was collected in online panels in Argentina and Germany, targeting adults fluent in Spanish or German respectively, using quotas for age and sex to resemble the joint marginal distribution in the general population. By selecting a general population sample, we ensured a mixture of different health states. This approach increases the likelihood of including individuals across the full spectrum of physical functioning, including those at the high-functioning end. Individuals who do not speak the language of administration and those unable to consent were excluded. Data was collected by a social research institute (Cint Deutschland GmbH). Comparable data from English-speaking subjects were already collected as part of the research project that developed the extended item bank through a US-based market research firm (Opinions for Good [Op4G]).

Besides PF, we collected sociodemographic variables and assessed overall health status of the participants with four items from the PROMIS Global Health Physical and Mental 2a two-item short forms [7]. The Physical Health short form consists of items Global03 (In general, how would you rate your physical health?) and Global06 (To what extent are you able to carry out your everyday physical activities such as walking, climbing stairs, carrying groceries, or moving a chair?). The Global Mental Health items are Global04 (In general, how would you rate your mental health, including your mood and your ability to think?) and Global05 (In general, how would you rate your satisfaction with your social activities and relationships?). The PROMIS Global Health measures were not collected in the USA.

### Unidimensionality

The items of a test are considered as unidimensional if they all measure the same, single, latent construct, in this case *physical function*. To evaluate the theoretical assumption of unidimensionality of the construct and to establish the foundations for using Item Response Theory (IRT) models, we performed a confirmatory factor analysis (CFA), a graded response model (GRM), and an exploratory bifactor model. This model decomposes item variance into a general factor and specific factors. We reported Explained Common Variance (ECV), the ratio of the general factor eigenvalue to the sum of all eigenvalues, which indicates unidimensionality. Additionally, we estimated coefficient omega (omega H) to assess the general factor saturation of the test [29].

We used the following fit statistics and thresholds to indicate good model fit: root mean squared error of approximation (RMSEA) < 0.06, standardized root means square residual (SRMR) ≤ 0.08, comparative fit index (CFI) ≥ 0.95, and Tucker-Lewis index (TLI) ≥ 0.95 [17]. To determine how well a unidimensional graded response model fitted the data, the M2* test statistic was calculated [3]. As suggested by Reise et al. (2013), we used the explained common variance by the general factor (ECV, cut-off < 0.6) as well as the coefficient omega hierarchical (OmegaH, cut-off > 0.8) as additional indicators of sufficient unidimensionality.

### Monotonicity

Monotonicity refers to a consistent, non-decreasing relationship between individual item scores and the levels of the underlying construct they measure. If one respondent scores higher on a specific item than another, their total score on the assessment should reflect this by not being lower than the score of the second respondent. For our analysis, we adopt the threshold of Loevinger's *H* values greater than 0.3 as an indicator of monotonicity, following the guidelines suggested by Sijtsma and Molenaar [20], which compares the number of violations to this pattern to the number that would be expected in a set of unrelated items [25].

### Item independence

The assumption of independence posits that the relationship between any two items is solely mediated by the construct they measure. To examine this, we used Yen's Q3 residual covariance statistic, adopting a criterion where values greater than 0.2 signal the presence of local dependence between items, as noted by [5]. Elevated residual covariance implies that responses to one item might influence responses to another or that both items are capturing an additional, unintended construct.

### Measurement invariance

Measurement invariance refers to the stability of the relationship between item responses and levels of the physical function, irrespective of population subgroup, such as countries [24]. Violations of measurement invariance indicate differential item functioning (DIF), a phenomenon that can skew the interpretation of an item's measurement across diverse contexts and lead to bias.

To illustrate, consider an item that asks about difficulties encountered when using public transportation. At similar levels of physical function, respondents from countries with well-developed public transportation systems, like Japan or Germany, might report fewer difficulties compared to respondents from countries where public transport systems are less accessible, such as in some rural areas of the United States. Hence, the item does not equally measure physical function across different country contexts, but rather reflects differences in infrastructure, accessibility, and culture related to transport e.g., quality, use, and access to public transport.

DIF can manifest in two distinct forms: uniform and non-uniform. Uniform DIF occurs when a specific comparison group (e.g., respondents from a given country) consistently shows a higher or lower likelihood of selecting responses across all levels of the underlying trait. Non-uniform DIF, however, occurs when the impact of the underlying trait on the likelihood of selecting a certain response category differs across groups. This means that the relationship between the trait level and the probability of a particular response is not consistent across groups. For example, at lower levels of physical function, respondents from one group might be more likely to choose certain categories compared to another group, but this pattern might change at higher levels of physical function. This variation can indicate that different groups interpret or value the items differently depending on their trait levels, which can result in differentiated item slopes for each group [24]. Non-uniform DIF is therefore characterized by an interaction between group membership and trait level in predicting responses.

In our analyses, we investigated DIF using the ordinal logistic regression framework [4]. This method compares the fit of different ordinal logistic regression models to predict item responses to an item based on the latent construct. If DIF exists, the addition of the covariate of interest (e.g. country) improves model. Using this framework, a main effect for the covariate is indicative of uniform DIF, while the interaction between the conditioning score and the covariate would represent non-uniform DIF.

Within this framework a wide range of plausible analysis strategies are possible and analytic choices and decisions can influence the results and conclusions drawn from the analysis. To ensure the robustness of our findings, we decided to conduct a multiverse analysis approach to include all plausible choices [19, 21–23]. This novel approach, which we term 'Multiverse DIF analyses', involves systematically varying the analytic choices across the entire range of plausible options and examining how these choices affect which items are flagged for DIF.

Specifically, we varied the following factors:

1. the country comparison (so we compared either all three countries simultaneously, or compared USA with Argentina, USA with Germany, or Argentina with Germany);
2. whether or not to include age as predictor in the ordinal regression models, as the Argentinian sample was on average nine years younger. Age was included as a linear effect and an interaction term with country to account for potential differences in how age affects physical function across countries;
3. the parameters for the Item Response Theory (IRT) model to estimate the latent trait (either using established PROMIS parameters or estimating parameters from the data at hand using a multigroup GRM);
4. the detection criterion for DIF, including likelihood ratio tests (LRT), LRT with Bonferroni correction, LRT with Benjamini–Hochberg correction, change in beta, and pseudo *R*^*2*^ values (Cox-Snell, Nagelkerke, McFadden); and
5. the respective flagging criteria, meaning the threshold for determining the presence of DIF, with different values for LR (0.02, 0.03, 0.05), Beta (0.01, 0.05), and *R*^*2*^ (2%, 3%, 5%).

Overall, this gives 272 unique combinations. We assessed the frequency with which each item was flagged across all analytical strategies. This approach enabled us to pinpoint specific items that consistently exhibited DIF and to identify which analytic decisions led to significantly divergent outcomes.

### Impact of DIF

To comprehensively assess the potential impact of DIF at the item level, we employed a visualization strategy. We compared models ignoring and accounting for DIF between languages, using Bland–Altman Plots to compare T-Scores across the spectrum of PF. We also compared the overall distribution of T-Scores in each sample and assessed the test characteristic curve.

### Open science practices

All data and R code for reproducible data analysis can be found at the Open Science Framework (https://osf.io/c75qv/). As PROMIS item parameters are proprietary, we followed recommendations to perturbate item parameters [10].

## Results

### Descriptives

See Table 1 for the information on demographic characteristics of the three countries, and on the distribution of the PROMIS Physical Function and PROMIS Global Health. Furthermore, Fig. S1 displays the item responses to the 35 new items across the three countries.

**Table 1:** Summary statistics of sociodemographic information and PROMIS Measures

Sociodemographic factors	USA (N = 1600)	Germany (N = 1000)	Argentina (N = 1001)
Age			
Min	18	18	18
Max	88	69	69
Mean (sd)	44.27, 16.15	44.93 (14.54)	35.58 (11.84)
Median	43	46	34
Gender, female			
N (%)	926 (58)	513 (51)	489 (49)
Education			
Basic education	115 (7)	77 (8)	284 (28)
Secondary Education	398 (25)	395 (40)	367 (37)
Vocational/Some College	459 (29)	293 (29)	242 (24)
Higher Education	628 (39)	235 (24)	108 (11)
PROMIS physical function			
Min	18.99	18.99	24.1
Floor (%)	12 (1)	6 (1)	1 (0)
Max	74.98	74.98	74.98
Ceiling (%)	90 (6)	27 (3)	21 (2)
IQR	15.78	13.04	9.34
Mean (sd)	50.27 (12.26)	51.37 (10.28)	52.77 (7.84)
Median	50.86	52.02	52.46
PROMIS Global Physical Health			
Mean (sd)	–	48.27 (7.90)	50.04 (8.03)
Median	–	50	50
PROMIS Global Mental Health			
Mean (sd)	–	47.65 (7.81)	50.57 (7.54)
Median	–	48.60	48.60

Floor/Ceiling: Number of individuals who reached the floor/ceiling value, followed by the percentage of the total sample. All PROMIS measures are reported as T-Scores with a mean of 50 and a standard deviation of 10 in the general population. We assessed PROMIS Global Mental and Physical Health with the PROMIS Global Health Physical and Mental 2a two-item short forms [7]

### Unidimensionality

Results of the unidimensionality assessment are shown in Table 2. Fit indices of the CFA and the GRM showed violations of the unidimensionality assumption. Fit indices varied across countries, with the USA showing the most favorable estimates. An exploratory bifactor model suggested a predominantly unidimensional structure of the data given the ECV. Given this evidence, along with the fact that the established PROMIS Physical Function measurement model is unidimensional, our further analysis was conducted assuming unidimensionality.

**Table 2:** Unidimensionality testing based on CFA, GRM, and bifactor model

	a) CFA			b) GRM							c) Bifactor model					
Country	CFI_r_^a^	TLI_r_^b^	RMSEA_r_	M2*	*df*	*p*	CFI^a^	TLI^b^	SRMR^c^	RMSEA^d^	RMSEAr^d^	CFI_r_^a^	TFI_r_^b^	SRMR^c^	ECV^e^	OmegaH^f^
All Countries	0.838	0.828	0.106 [0.104, 0.107]	11,572.63	455	< 0.01	0.906	0.899	0.044	0.082 [0.081, 0.084]	0.065 [0.064, 0.067]	0.927	0.917	0.031	0.810	0.872
USA	0.890	0.883	0.095 [0.093, 0.097]	2,889.86	455	< 0.01	0.964	0.961	0.035	0.058 [0.056, 0.06]	0.056 [0.053, 0.058]	0.955	0.948	0.027	0.828	0.878
Argentina	0.737	0.720	0.114 [0.111, 0.117]	2,683.60	455	< 0.01	0.866	0.856	0.065	0.07 [0.067, 0.073]	0.069 [0.066, 0.072]	0.878	0.860	0.049	0.663	0.752
Germany	0.761	0.746	0.135 [0.132, 0.138]	5,293.49	455	< 0.01	0.852	0.841	0.054	0.103 [0.101, 0.106]	0.078, [0.075, 0.080]	0.902	0.888	0.041	0.781	0.854

^a^A CFI value of 0.95 or larger is considered to indicate good model fit

^b^A TLI value of 0.95 or larger is considered to indicate good model fit

^c^A SRMR value of 0.08 or smaller is considered to indicate appropriate model fit

^d^An RMSEA value of 0.08 or smaller is considered to indicate appropriate model fit

^e^An ECV value of 0.6 or larger is considered to indicate sufficient unidimensionality of a model

^f^An OmegaH value of 0.8 or larger is considered to indicate sufficient unidimensionality of a model

*CFI* comparative fit index, *CFI*_*r*_ CFI robust, *TLI* Tucker-Lewis index, *TLI*_*r*_ Tucker-Lewis index robust, *SRMR* standardized root means square residual; Brackets indicate 90% confidence interval, *RMSEA* root mean square error of approximation, *RMSEA*_*r*_ robust root mean square error of approximation; *p p*-value of M2

### Monotonicity

All items in each scale showed Loevinger’s H statistics > 0.3, with an overall Scale H = 0.601, SE = 0.007, suggesting monotonicity.

### Item independence

In total, 22 item pairs out of 595 unique covariances (< 5%) showed a higher residual covariance statistic Yen’s Q3 than 0.20. The highest residual correlation was 0.48 between PFM38 (Are you able to lift and load one 50-pound (25 kg) bag of sand into a car?) and PFM 44 (Are you able to carry a 50 lb (25 kg) bag of sand 25 yards (25 m)?). Given that some residual covariance should be expected to occur even by chance, and that so few potential item doublets occurred, these results are broadly supportive of the local independence assumption necessary to proceed with IRT modeling.

### Differential item functioning

#### Multiverse differential item functioning (DIF) analysis

**Fig. 1:**
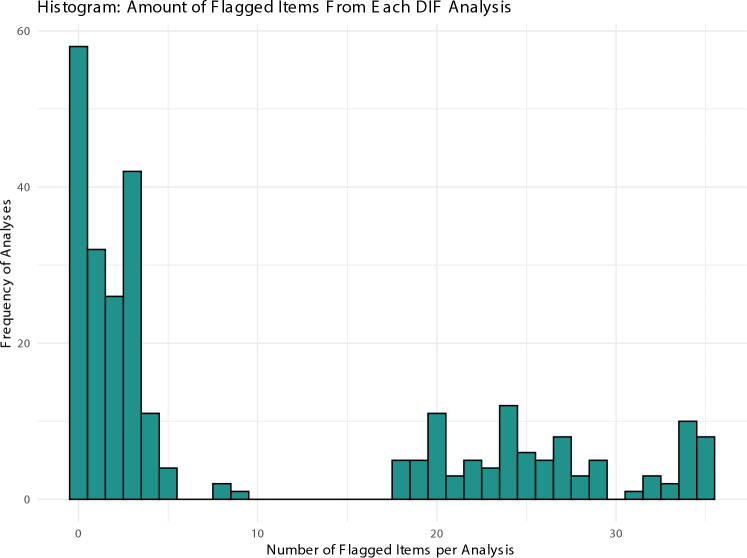
Histogram for number of flagged items from each DIF analysis. This histogram captures the range of outcomes from multiple Differential Item Functioning (DIF) analyses. Each bar represents the frequency of analyses that flagged a certain number of items, with the x-axis specifying the number of items flagged and the y-axis depicting the count of analyses

In our multiverse DIF analysis, we conducted a total of 272 DIF analyses. Figure 1 shows the amount of DIF detected varies greatly between analyses—from zero to all 35 items being flagged. The histogram highlights the skewness towards analyses that identified a fewer number of items, with a noticeable concentration in the 0–5 item range. The right tail of the histogram, which includes analyses flagging more than ten items, is exclusively composed of analyses using Likelihood Ratio-based criteria (incorporating both Bonferroni and Benjamini–Hochberg corrections for multiple testing).

**Fig. 2:**
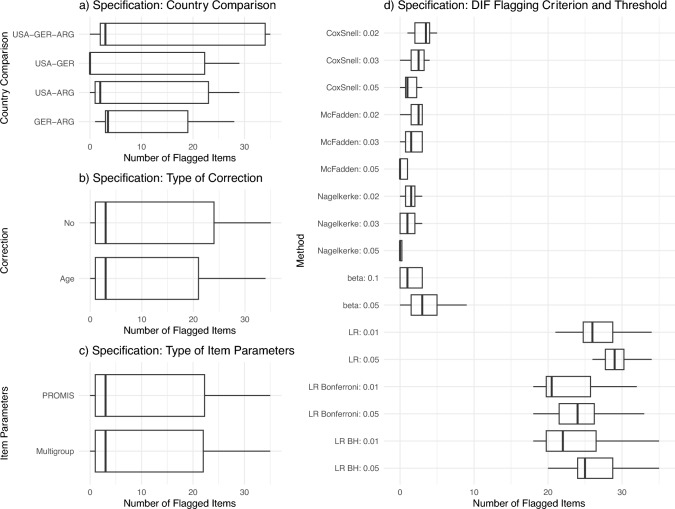
Boxplots of the multiverse for each specification. Boxplots representing the results of Differential Item Functioning (DIF) analyses. Each point is an individual DIF analysis. Panel **a** shows country comparisons. Panel **b** explores the impact of age adjustment. Panel **c** depicts the number of flagged items using different item parameters. Panel **d** presents a range of DIF flagging criteria and thresholds

Figure 2 revealed systematic patterns in these results. Panel a) illustrated a notable trend where the comparisons between the US and Germany yielded minimal DIF items, indicated by the median's proximity to zero and conversely that the Germany-Argentina comparison frequently identified multiple DIF items. Simultaneously, Panels b) and c) suggest that whether correcting for age differences or choosing different item parameters only has a marginal effect on the number of flagged DIF items. Panel d) shows that LRT methods were much more sensitive to identify DIF. Compared to all other methods, LRT (with/without adjustment for multiple testing) were highly sensitive against item parameter differences and flagged a high number of items for DIF. The pseudo-*R*^2^ estimators and beta coefficients exhibited variable sensitivity, ranging from identifying negligible to a moderate number of DIF items, hinting at a more graduated approach to flagging potential DIF items. See Table 3 for the percentages of flagged items for each specification.

**Table 3:** Percentage of flagged items based on all specifications within the DIF multiverse

Specification	Items flagged	Flagged items LR excluded (%)	Flagged items all methods (%)
(a) Country comparison			
Germany-Argentina	663	2.2	7.0
USA-Argentina	641	1.1	6.7
USA-Germany	588	0.1	6.2
USA-Germany-Argentina	903	1.5	9.5
(b) Adjustment			
For Age	1341	2.0	14.1
No adjustment	1454	2.8	15.3
(c) Item parameters			
Estimated with multigroup	1399	2.4	14.7
PROMIS	1396	2.4	14.7
(d) Flagging Criterion			
R^2^ Cox	106	1.7	1.1
R^2^ McFadden	67	1.1	0.7
R^2^ Nagelkerke	48	0.8	0.5
Beta	78	1.3	0.8
LRT	2496	–	26.2

Items flagged: Total number of times an item was flagged in all 272 DIF analyses; Percentages items were flagged based on different specifications for (a) country comparisons, (b) correction for age differences, c) different item parameters and d) different flagging criteria. Flagged items based on all methods, including likelihood ratio tests (LRT), are included as a point of reference, but our interpretation focusses on methods except the LRT method

During our examination of the remaining 176 DIF assessments without the LRT-based methods,^1^ we identified DIF disproportionally often (applying a post hoc cutoff of more than 10% of analyses) in four specific items: Item **PFM46** (“Are you able to pull a sled or a wagon with two children (total 100 lbs/50 kg) for 100 yards (100 m)?”) was flagged in 60.2% of these analyses, item **PFM33** (“Are you able to walk across a balance beam?”) in 52.8%, item **PFM16** („Are you able to pass a 20-pound (10 kg) turkey or ham to other people at the table? “) in 34.1%, and item **PFM51** (“Are you able to swim laps for 30 min at a moderate pace?") in 10.2%. A comprehensive overview of all items can be found in Table 4, revealing that 22 items were never flagged, and 9 items were flagged between 1 and 5 times. See Fig. S2a–d for a comprehensive visualization showing under which combination of analytic decisions the respective items showed DIF.

**Table 4:** Times individual items were flagged for DIF based on Pseudo R^2^ and beta

Item ID	Item stem	k	Percent
**PFM46**	**Are you able to pull a sled or a wagon with two children (total 100 lbs/50 kg) for 100 yards (100 m)?**	**106**	**60.2**
**PFM33**	**Are you able to walk across a balance beam?**	**93**	**52.8**
**PFM16**	**Are you able to pass a 20-pound (10 kg) turkey or ham to other people at the table?**	**60**	**34.1**
**PFM51**	**Are you able to swim laps for 30 min at a moderate pace?**	**18**	**10.2**
PFM40	Are you able to climb a 6-foot (2 m) ladder?	5	2.8
PFM12	Are you able to lift a heavy object (20 lbs/10 kg) above your head?	4	2.3
PFM15	Are you able to hit the backboard with a basketball from the free-throw line (13 ft/4 m)?	3	1.7
PFM26	Are you able to make sharp turns while running fast?	3	1.7
PFM38	Are you able to lift and load one 50-pound (25 kg) bag of sand into a car?	2	1.1
PFM44	Are you able to carry a 50 lb (25 kg) bag of sand 25 yards (25 m)?	2	1.1
PFM1	Are you able to dig a 2-foot (1/2 m) deep hole in the dirt with a shovel?	1	0.6
PFM25	Are you able to come to a complete stop while running?	1	0.6
PFM43	Are you able to push an empty refrigerator forward 1 yard (1 m)?	1	0.6
PFM2	Are you able to lift a heavy painting or picture to hang on your wall above eye-level?	0	0
PFM3	Are you able to paint the walls of a room with a brush or roller for 2 h without stopping to rest?	0	0
PFM4	Are you able to row a boat for 30 min without stopping to rest?	0	0
PFM6	Are you able to hand wash and wax a car for 2 h without stopping to rest?	0	0
PFM7	Are you able to complete 5 push-ups without stopping?	0	0
PFM9	Are you able to rake leaves or sweep for an hour without stopping to rest?	0	0
PFM10	Are you able to do a pull-up?	0	0
PFM17	Are you able to remove a heavy suitcase (50 lbs/25 kg) from an overhead bin on an airplane or bus?	0	0
PFM18	Are you able to continuously swing a baseball bat or tennis racket back and forth for 5 min?	0	0
PFM19	Are you able to complete 10 sit-ups without stopping?	0	0
PFM21	Are you able to climb the stairs of a 10-story building without stopping?	0	0
PFM23	Are you able to walk briskly for 20 min without stopping to rest?	0	0
PFM27	Are you able to jump rope for 10 min without stopping?	0	0
PFM28	Are you able to jump over an object that is 1 foot (30 cm) tall?	0	0
PFM29	Are you able to jump over a puddle that is 3 feet (1 m) wide?	0	0
PFM32	Are you able to jump 2 feet (60 cm) high?	0	0
PFM34	Are you able to stand on one foot with your eyes closed for 30 s?	0	0
PFM35	Are you able to walk in a straight line putting one foot in front of the other (heel to toe) for 5 yards (5 m)?	0	0
PFM36	Are you able to put your hands flat on the floor with both feet flat on the ground?	0	0
PFM37	Are you able to carry a large baby (15 lbs/7 kg) out of the house to a car or taxi?	0	0
PFM49	Are you able to stand up from a push-up position five times quickly?	0	0
PFM53	Are you able to dance energetically for an hour?	0	0

*k* = Number of times an item was flagged for DIF

Bold = Items flagged in > 10% of analyses

#### Influence of flagged DIF items at the test level

**Fig. 3:**
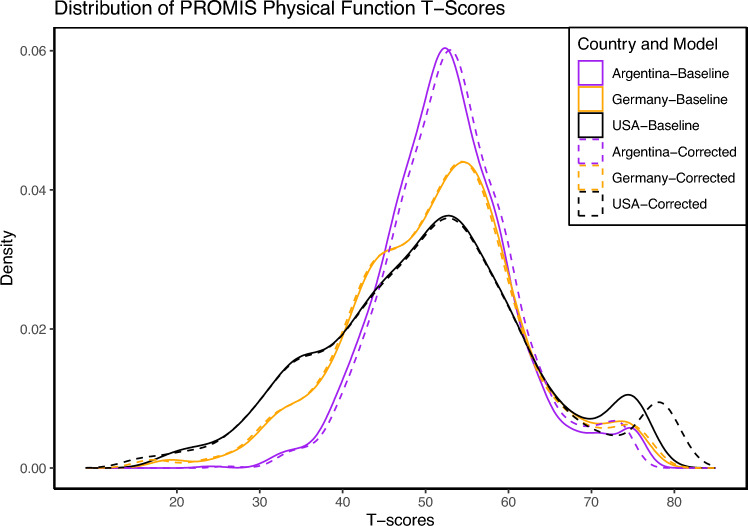
Distribution of PROMIS Physical Function T-Scores for Fully-Invariant and Partially-Invariant Models. Estimated density plots of physical function levels (T-Scores) for study participants from the USA, Germany, and Argentina. Solid lines represent the baseline (fully-invariant) model, and dashed lines represent the corrected (partially-invariant) models where item parameters for DIF items were estimated freely

Overall, correcting for DIF had only a small impact on the overall distribution of T-Scores, see Fig. 3 for a comparison of T-Scores between the fully-invariant model, which assumes that item parameters are identical across countries, and a partially-invariant model, which estimates item parameters freely for items that were flagged for DIF in > 10% of the multiverse DIF analyses (PFM16, PFM33, PFM46, and PFM 51). We used a Bland–Altman plot to further assess the agreement of individual scores. Specifically, this plot illustrate the difference between the T-Scores obtained by the two models against the average of those measurements. Ideally, if both methods are in perfect agreement, the differences should be randomly scattered around zero, showing no systematic bias.

**Fig. 4:**
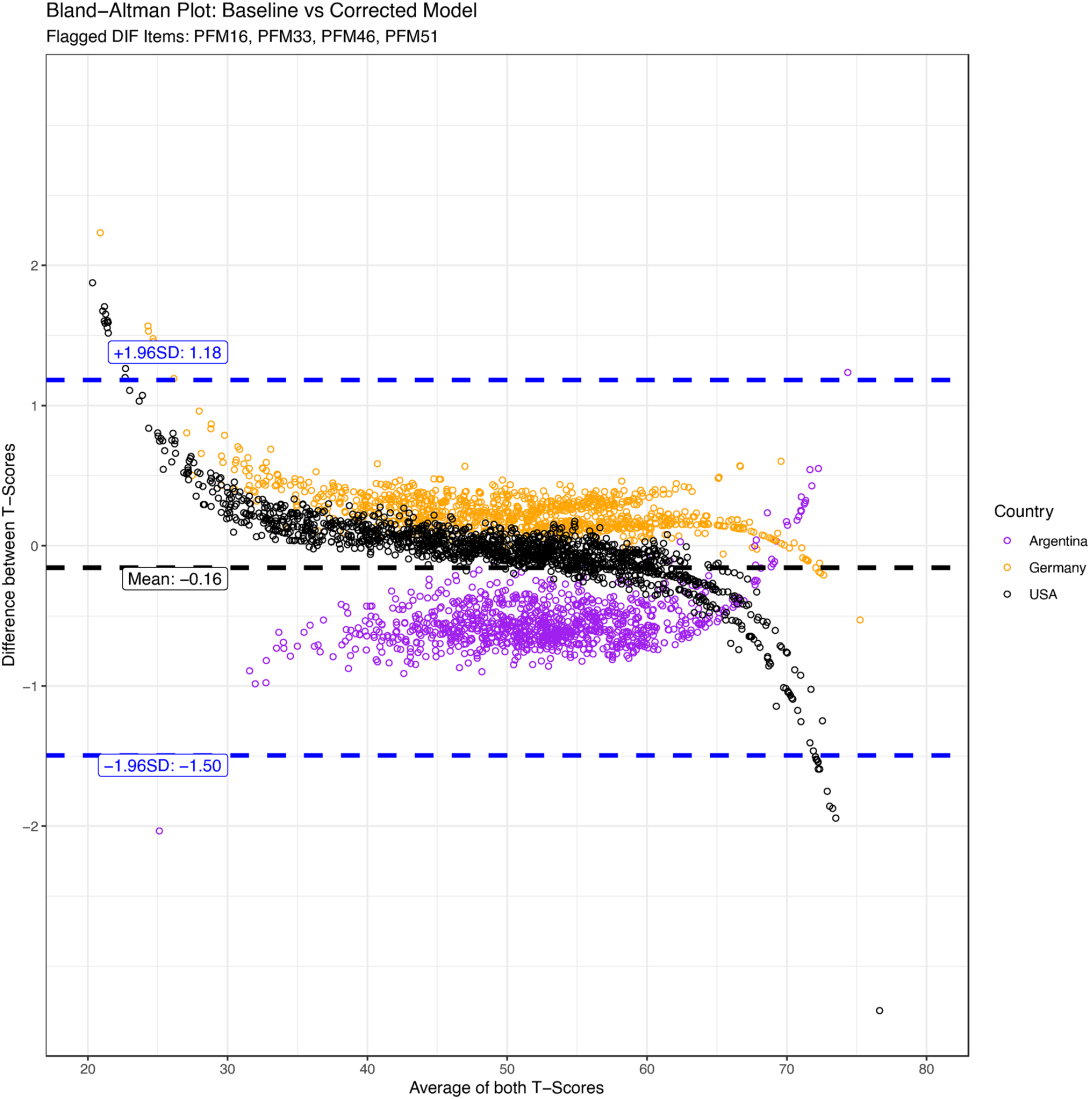
Bland–Altman Plots for the Comparison Between Fully-Invariant and Partially-Invariant Model. The black dotted line represents the mean difference between T-scores, the blue lines the upper and lower limits of agreement. If the methods would yield perfectly aligned results, the points in each plot would be expected to randomly jitter within the limits of agreement. (Color figure online)

We found a negligible mean difference of − 0.16 T-Scores between the partially-invariant and the fully-invariant model. 95% of differences were between − 1.50 and 1.18, indicating that even on an individual level, the model difference is small. The Bland–Altman plot analysis reveals a discernible pattern of agreement between the fully-invariant and partially-invariant models across three countries (Fig. 4). Germany and the USA exhibit a more consistent and similar pattern, with the differences between the models' T-Scores clustering closely together. This suggests that for these countries, the fully-invariant and partially-invariant models yield more comparable scores. Initially, for lower average T-scores, both countries display a positive difference, indicating that the fully-invariant model scores are higher than those of the partially-invariant model. However, as the average T-scores increase, this trend reverses for the USA, with the differences becoming negative, pointing to the fully-invariant model producing lower scores than the partially-invariant model at higher T-scores.

In contrast, Argentina presents a notably different trend, with larger variability in the differences between the fully-invariant and partially-invariant model scores. The differences for Argentina start below the mean difference line for lower T-scores, suggesting the fully-invariant model scores are lower in this range. Yet, as the T-scores increase, these differences cross above the mean difference line, indicating higher scores from the fully-invariant model at the upper end of T-scores. This variable pattern of agreement suggests that the items flagged for DIF may have a more pronounced effect on the T-Scores in the Argentine sample. The patterns observed reflect the slightly different wider (US) respectively narrower (Argentia) distribution of T-Scores when accounting for DIF.

See Figs. S3 and S4 for additional Test Information Function plots and Test Characteristic Curves plots suggesting minor differences between the fully-invariant and partially-invariant models.

## Identification of uniform and non-uniform DIF in select PROMIS items

We thoroughly examined the four items that were identified in the multiverse DIF analysis—PFM16, PFM33, PFM46, and PFM51—and found uniform DIF for the first three items and non-uniform DIF for the last item.

The graphical analyses presented in Fig. S5 to S8 offer a detailed visualization of the DIF observed in specific PROMIS items across the USA, Germany, and Argentina. The item characteristic curves (ICCs) and item response functions shed light on how participants from these countries perceive and respond to items PFM16, PFM33, PFM46, and PFM51. Fig. S5 illustrates that item PFM16 exhibits uniform DIF across all countries, primarily at lower theta levels, suggesting a universal challenge in this item's interpretation at lower physical function levels. Similarly, item PFM33, as depicted in Fig. S6, reveals that the difference in ICCs between the USA and Germany is more pronounced at lower theta levels, whereas the difference between the USA and Argentina emerges at medium theta levels. This pattern underscores the nuanced ways in which physical function is conceptualized across these cultures. Fig. S7 showcases item PFM46, where the discrepancy in ICCs between Argentina and the other two countries is mainly at medium levels of theta, indicating a distinct perception of this item's difficulty or relevance in Argentina as compared to the USA and Germany. Lastly, Fig. S8 for item PFM51 highlights that differences in ICCs between the USA and Germany are notable at lower levels of theta, while the differences between the USA and Argentina become more prominent at medium theta levels and show non-uniform DIF.

Despite these observed differences, the lower-right graphs in each Fig. (S5-S8), which represent the absolute differences between the ICCs weighted by the score distribution for the reference group (the USA), consistently indicate a minimal overall impact. This suggests that while there are country-specific differences in how certain items are perceived or answered, these do not substantially alter the test's ability to measure physical function uniformly across these diverse populations.

**Fig. 5:**
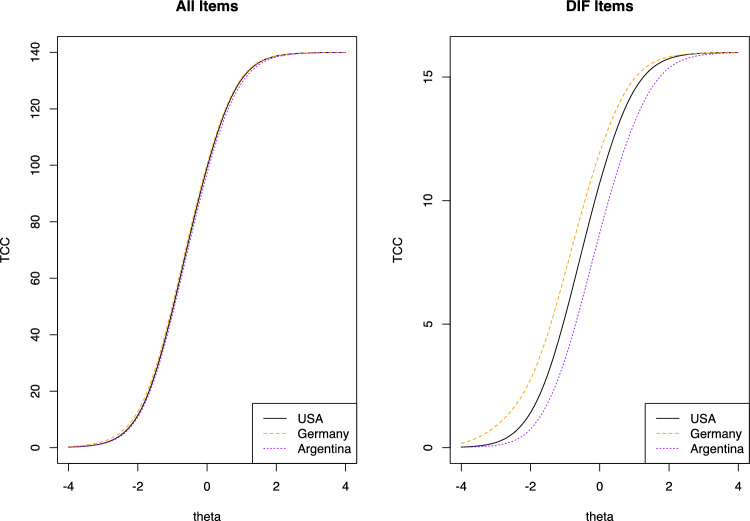
Impact of DIF items on test characteristic curves. These graphs show test characteristic curves (TCCs) for USA (black), Germany (orange dashed), and Argentina (purple dotted) using demographic–specific item parameter estimates. TCCs show the expected total scores for groups of items at each physical function level (theta). The graph on the left shows these curves for all of the items (both items with and without DIF), while the graph on the right shows these curves for the subset of these items found to have DIF. These curves suggest that at the overall test level there are some differences in the total expected score for individuals from all three countries. At the same level of ability (i.e., the same theta score on the x-axis), individuals from Germany obtained higher expected observed sum scores than while at the same level of ability individuals from Argentina had lower observed sum scores than the USA. (Color figure online)

Figure 5 suggests that at the overall test level there are negligible differences in the total expected score for individuals from all three countries. For all DIF items combined, respondents (given the same PF) from Germany have slightly higher expected item scores and respondents from Argentina have lower expected scores compared to the US reference group. In turn, this means PF estimates based on the US parameter would underestimate PF in Germany, whereas in Argentina it would overestimate true PF. See supplementary Table S1 for the partially-invariant and country specific item parameters for all identified items exhibiting DIF.

## Discussion

### Main findings

In this study, we translated and validated new ceiling items from the PROMIS Physical Functioning item bank into German and Spanish for use in Germany, Argentina, and the USA. We identified four items that were consistently susceptible to DIF across various analytic scenarios. The influence of these items on test-level scores was minimal, but could be more significant in Computerized Adaptive Testing (CAT) applications or when creating tailored short forms. In CATs, DIF can disrupt the algorithm's ability to select the most appropriate subsequent items, potentially leading to inaccurate or unfair assessments. For custom short forms, DIF in even a single item can substantially skew the results, as each item carries more weight in the overall scoring due to the brevity of the form. Hence, we recommend to either use country specific item parameters, or to omit these 4 items in CATs or short forms.

A critical aspect of our DIF analysis was the selection of the DIF flagging criterion and threshold. Analytic decisions besides the age adjustment, choice of item parameters, and country comparisons were similarly influential as the selection of the flagging method. For example, the use of Nagelkerke R^2^ with a threshold of 0.05 flagged 0.1% of items, Beta with 0.05 flagged 1% of items, while each individual LRT method flagged more than 5% of items. To assist researchers in navigating these complex decisions, we developed a 'lordif' wrapper function that calculates and visually represents the potential impact of different estimators on DIF flagging (https://github.com/cyplessen/lordifMultiverse). This tool provides a valuable resource for researchers to determine the robustness of their analytic decisions and the likelihood of items being flagged across a range of scenarios.

The robustness of our identified DIF items was supported by the stability of findings across multiple reasonable analytic decisions, which reinforces the identification of items sensitive to DIF. The impact of age differences between countries and the choice of item parameters were negligible factors in DIF detection. However, the variation in DIF-flagged items between country comparisons highlighted that cultural and linguistic differences might affect item perception differently, emphasizing the need for careful consideration in multinational research contexts.

### Content based explanation of different item properties

The DIF observed in various physical and sports-related items among respondents from the USA, Germany, and Argentina could be attributed to the cultural context, traditional practices, and varying degrees of exposure and familiarity with the activities in question. For instance, the sled or wagon pulling item (PFM 46) might highlight the influence of specific cultural and recreational activities prevalent in each country. In the USA, where activities involving sleds or wagons may be traditional, respondents are likely to find these scenarios more relatable and manageable. Similarly, the item about passing a large turkey or ham at the table (PFM 16) resonates differently across cultures, with the USA having a unique connection to this activity through US Thanksgiving traditions. The balance beam (PFM 33) and swimming laps items (PFM 51) also demonstrate DIF, reflecting variations in physical education curriculums, access to facilities, and cultural attitudes towards fitness and physical challenges. While regions in the USA and Germany might emphasize activities that develop balance and swimming skills, making these tasks seem more feasible, such emphasis might be less in Argentina, or the infrastructure might not support regular participation in these activities.

### Strengths and limitations

Our study demonstrates considerable robustness through the sensitivity of our findings across a variety of reasonable analytic decisions. The identification of uniform DIF in specific items, notably PFM16, PFM33, PFM46, and non-uniform DIF for item PFM51, underscores the meticulous nature of our analytical approach. Furthermore, the minimal impact of DIF on the test characteristic curves and the universal applicability of PROMIS items across diverse populations from Argentina, Germany, and the U.S. highlight the global relevance and adaptability of the PROMIS initiative. Moreover, our detailed item-level analysis offers in-depth insights into how each item functions across different cultural contexts, providing a strong foundation for future refinements of the PROMIS item bank and the creation of culturally sensitive assessment tools.

However, our study is not without its limitations. The consistent flagging of all items for DIF using the LRT method raises concerns about its oversensitivity and questions its practical utility in discerning meaningful differences. Moreover, our analysis was confined to the lordif framework, not extending to other established methods for DIF analysis such as multiple-group confirmatory factor analysis, other IRT-based methods, Mantel–Haenszel procedures, or the Rasch Model Comparison Test [27, 28].

The possibility of multidimensionality within the Physical Functioning item bank due to its inclusion of various subdomains might have led to suboptimal unidimensionality measures, impacting the precise assessment of physical functioning. Additionally, the comprehensiveness of our analyses was limited by the absence of certain variables in the U.S. sample that were present in the datasets from Germany and Argentina. While this restricted the scope of our comparisons, it did not impact the validity of the results obtained. Lastly, the substantial age difference, with the Argentinian sample being on average 10 years younger than the U.S. and German samples, could have implications for the interpretation of DIF and the generalizability of the findings across these populations—even though our multiverse analysis indicated that age differences were not a relevant source of DIF as corrections for these age differences had only a minimal impact on the results.

## Overall conclusion

Our analysis supports the universal applicability of the PROMIS physical functioning items across populations in Argentina, Germany, and the U.S. Despite the identification of DIF in some items, the overall impact on test scores is negligible, and the test characteristics remain robust. However, slight variations in scores after correcting for DIF—lower for Germany and higher for Argentina compared to the U.S.—highlight the subtleties of cross-cultural measurement and the need for ongoing evaluation. In multinational studies, the exclusion of DIF-affected items or the use of country-specific item parameters and/or group hyperparameters may be necessary for the optimal administration of computer adaptive tests and the formulation of tailored short forms. We have provided such corrected and country-specific item parameters for all identified items exhibiting DIF in this study (Table S1). The study's multiverse DIF analysis approach, accounting for age and country specific factors, provides a strong foundation for the PROMIS items' use, indicating that they maintain their validity and reliability across different countries and cultural contexts.

## Supplementary Information

Below is the link to the electronic supplementary material.

Supplementary file1 (DOCX 894 KB)

## Footnotes

1 Notably, most items were consistently flagged in analysis using the LR method (74% of items), leading us to exclude this estimator from our figures and the results section for clarity. These methods, even after correcting for multiple testing and false discovery rates, tend to produce type 1 errors in highly powered contexts.

## References

[1] Beauchamp, M. K., Hao, Q., Kuspinar, A., Amuthavalli Thiyagarajan, J., Mikton, C., Diaz, T., & Raina, P. (2023). A unified framework for the measurement of mobility in older persons. *Age and Ageing,**52*(4), 82–85. 10.1093/ageing/afad125 10.1093/ageing/afad125 PMC10615050 37902518

[2] Bruce, B., Fries, J., Lingala, B., Hussain, Y. N., & Krishnan, E. (2013). Development and assessment of floor and ceiling items for the PROMIS physical function item bank. *Arthritis Research & Therapy,**15*(5), R144. 10.1186/ar4327 24286166 10.1186/ar4327 PMC3978724

[3] Cai, L., & Hansen, M. (2013). Limited-information goodness-of-fit testing of hierarchical item factor models. *British Journal of Mathematical and Statistical Psychology,**66*(2), 245–276. 22642552 10.1111/j.2044-8317.2012.02050.x PMC3760206

[4] Choi, S. W., Gibbons, L. E., & Crane, P. K. (2011). lordif: An R package for detecting differential item functioning using iterative hybrid ordinal logistic regression/item response theory and Monte Carlo simulations. *Journal of Statistical Software,**39*(8), 1–30. 21572908 10.18637/jss.v039.i08 PMC3093114

[5] Christensen, K. B., Makransky, G., & Horton, M. (2017). Critical values for Yen’s Q3: Identification of local dependence in the Rasch model using residual correlations. *Applied Psychological Measurement,**41*(3), 178–194. 10.1177/0146621616677520 29881087 10.1177/0146621616677520 PMC5978551

[6] Fries, J. F., Lingala, B., Siemons, L., Glas, C. A. W., Cella, D., Hussain, Y. N., Bruce, B., & Krishnan, E. (2014). Extending the floor and the ceiling for assessment of physical function: Extended floor and ceiling assessment of physical function. *Arthritis & Rheumatology,**66*(5), 1378–1387. 10.1002/art.38342 24782194 10.1002/art.38342 PMC4012831

[7] Hays, R. D., Schalet, B. D., Spritzer, K. L., & Cella, D. (2017). Two-item PROMIS® global physical and mental health scales. *Journal of Patient-Reported Outcomes,**1*(1), 2. 10.1186/s41687-017-0003-8 29757325 10.1186/s41687-017-0003-8 PMC5934936

[8] Hays, R. D., Spritzer, K. L., Amtmann, D., Lai, J.-S., Dewitt, E. M., Rothrock, N., Dewalt, D. A., Riley, W. T., Fries, J. F., & Krishnan, E. (2013). Upper-extremity and mobility subdomains from the Patient-Reported Outcomes Measurement Information System (PROMIS) adult physical functioning item bank. *Archives of Physical Medicine and Rehabilitation,**94*(11), 2291–2296. 10.1016/j.apmr.2013.05.014 23751290 10.1016/j.apmr.2013.05.014 PMC3812258

[9] Kaat, A. J., Schalet, B. D., Rutsohn, J., Jensen, R. E., & Cella, D. (2018). Physical function metric over measure: An illustration with the Patient-Reported Outcomes Measurement Information System (PROMIS) and the Functional Assessment of Cancer Therapy (FACT): Linking PROMIS PF and FACT-G PWB. *Cancer,**124*(1), 153–160. 10.1002/cncr.30981 28885707 10.1002/cncr.30981 PMC5734956

[10] Mansolf, M., Lai, J.-S., & Cella, D. (2023). Using parameter perturbation to facilitate transparency in measure development. *Quality of Life Research*. 10.1007/s11136-023-03572-1 38070033 10.1007/s11136-023-03572-1 PMC13098858

[11] Meade, A. W. (2010). A taxonomy of effect size measures for the differential functioning of items and scales. *Journal of Applied Psychology,**95*(4), 728–743. 10.1037/a0018966 20604592 10.1037/a0018966

[12] Millsap, R. E., & Everson, H. T. (1993). Methodology review: Statistical approaches for assessing measurement bias. *Applied Psychological Measurement,**17*(4), 297–334. 10.1177/014662169301700401

[13] Patient-Reported Outcomes Measurement Information System. (2013). *PROMIS® Instrument Development and Validation Scientific Standards, Version 2.0, (revised May 2013)*. http://www.healthmeasures.net/images/PROMIS/PROMISStandards_Vers2.0_Final.pdf

[14] Rose, M., Bjorner, J. B., Gandek, B., Bruce, B., Fries, J. F., & Ware, J. E. (2014). The PROMIS Physical Function item bank was calibrated to a standardized metric and shown to improve measurement efficiency. *Journal of Clinical Epidemiology,**67*(5), 516–526. 10.1016/j.jclinepi.2013.10.024 24698295 10.1016/j.jclinepi.2013.10.024 PMC4465404

[15] Samejima, F. (2016). *Graded response models*. Chapman and Hall/CRC.

[16] Schalet, B. D., Kaat, A., Vrahas, M., Buckenmaier III, C., Barnhill, R., & Gershon, R. C. (2016). *Extending the ceiling of an item bank: Development of above-average physical function items for PROMIS*. *25*, 109–109

[17] Schreiber, J. B., Nora, A., Stage, F. K., Barlow, E. A., & King, J. (2006). Reporting structural equation modeling and confirmatory factor analysis results: A review. *The Journal of Educational Research,**99*(6), 323–338. 10.3200/JOER.99.6.323-338

[18] Scott, N. W., Fayers, P. M., Aaronson, N. K., Bottomley, A., de Graeff, A., Groenvold, M., Gundy, C., Koller, M., Petersen, M. A., Sprangers, M. A., the EORTC Quality of Life Group and the Quality of Life Cross-Cultural Meta-Analysis Group. (2010). Differential item functioning (DIF) analyses of health-related quality of life instruments using logistic regression. *Health and Quality of Life Outcomes,**8*(1), 81. 10.1186/1477-7525-8-81 20684767 10.1186/1477-7525-8-81 PMC2924271

[19] Shunsen, H., Haojie, C., Xiaoxiong, L., Xinran, A. I., & Yun, W. (2023). Multiverse-style analysis: Introduction and application. *Advances in Psychological Science,**31*(2), 196. 10.3724/SP.J.1042.2023.00196

[20] Sijtsma, K., & Molenaar, I. (2002). *Introduction to Nonparametric Item Response Theory*. SAGE Publications Inc.

[21] Simonsohn, U., Simmons, J. P., & Nelson, L. D. (2015). Specification Curve: Descriptive and inferential statistics on all reasonable specifications. *SSRN Electronic Journal,**11*, 1–18. 10.2139/ssrn.2694998

[22] Simonsohn, U., Simmons, J. P., & Nelson, L. D. (2020). Specification curve analysis. *Nature Human Behaviour,**4*(11), 1208–1214. 10.1038/s41562-020-0912-z 32719546 10.1038/s41562-020-0912-z

[23] Steegen, S., Tuerlinckx, F., Gelman, A., & Vanpaemel, W. (2016). Increasing transparency through a multiverse analysis. *Perspectives on Psychological Science,**11*(5), 702–712. 10.1177/1745691616658637 27694465 10.1177/1745691616658637

[24] Teresi, J. A., Wang, C., Kleinman, M., Jones, R. N., & Weiss, D. J. (2021). Differential item functioning analyses of the patient-reported outcomes measurement information system (PROMIS®) measures: Methods, challenges, advances, and future directions. *Psychometrika,**86*(3), 674–711. 10.1007/s11336-021-09775-0 34251615 10.1007/s11336-021-09775-0 PMC8889890

[25] van Schuur, W. H. (2003). Mokken scale analysis: Between the Guttman scale and parametric item response theory. *Political Analysis,**11*(2), 139–163. 10.1093/pan/mpg002

[26] Voshaar, M. O., Vonkeman, H. E., Courvoisier, D., Finckh, A., Gossec, L., Leung, Y. Y., Michaud, K., Pinheiro, G., Soriano, E., & Wulfraat, N. (2019). Towards standardized patient reported physical function outcome reporting: Linking ten commonly used questionnaires to a common metric. *Quality of Life Research,**28*(1), 187–197. 10.1007/s11136-018-2007-0 30317425 10.1007/s11136-018-2007-0 PMC6339672

[27] Woods, C. M., Cai, L., & Wang, M. (2013). The langer-improved wald test for DIF testing with multiple groups: Evaluation and comparison to two-group IRT. *Educational and Psychological Measurement,**73*(3), 532–547. 10.1177/0013164412464875

[28] Wu, H., & Estabrook, R. (2016). Identification of confirmatory factor analysis models of different levels of invariance for ordered categorical outcomes. *Psychometrika,**81*(4), 1014–1045. 10.1007/s11336-016-9506-0 27402166 10.1007/s11336-016-9506-0 PMC5458787

[29] Zinbarg, R. E., Revelle, W., Yovel, I., & Li, W. (2005). Cronbach’s α, Revelle’s β, and Mcdonald’s ωH: Their relations with each other and two alternative conceptualizations of reliability. *Psychometrika,**70*(1), 123–133. 10.1007/s11336-003-0974-7

