# Supplementary material for: Differential item functioning between English, German, and Spanish PROMIS® physical function ceiling items
Source: Qual Life Res. 2024 Dec 16;34(5):1377–91. doi: 10.1007/s11136-024-03866-y (PMC12064622; doi:10.1007/s11136-024-03866-y)
Supplement: Supplementary file 1 — Supplementary file1 (DOCX 894 KB) [file 11136_2024_3866_MOESM1_ESM.docx]

# Appendix

### **Fig. S1** Item Responses of all Included Ceiling Items

*Note*. This graph displays participants’ responses to the 35 new ceiling items across Argentina, Germany, and the US.

***Fig. S2*** *a*-d Which and How Factor Combinations for Individual Flagged Items

Fig. S2a

Note. The figure displays the results from a series of Differential Item Functioning (DIF) analyses for item PFM46 using a multiverse approach. Each specification number on the horizontal axis represents a unique combination of analytical factors, including country comparisons, age corrections, parameter estimation methods, and DIF detection criteria. The purple colour indicates the presence of DIF, while the orange colour indicates the absence of DIF for each specification. Item PFM46 *Are you able to pull a sled or a wagon with two children (total 100 lbs/50 kg) for 100 yards (100 m)?* was flagged for DIF in 60.2% of all 176 analyses.

Fig. S2b

*Note.* The figure displays the results from a series of Differential Item Functioning (DIF) analyses for item PFM16 using a multiverse approach. Each specification number on the horizontal axis represents a unique combination of analytical factors, including country comparisons, age corrections, parameter estimation methods, and DIF detection criteria. The purple colour indicates the presence of DIF, while the orange colour indicates the absence of DIF for each specification. Item PFM16 *Are you able to pass a 20-pound (10 kg) turkey or ham to other people at the table?* was flagged for DIF in 34.1% of all 176 analyses. Most often for the comparison between Germany and Argentina.

Fig. S2c

*Note.* The figure displays the results from a series of Differential Item Functioning (DIF) analyses for item PFM51 using a multiverse approach. Each specification number on the horizontal axis represents a unique combination of analytical factors, including country comparisons, age corrections, parameter estimation methods, and DIF detection criteria. The purple colour indicates the presence of DIF, while the orange colour indicates the absence of DIF for each specification. Item PFM51 *Are you able to swim laps for 30 minutes at a moderate pace?* was flagged for DIF in 10.2% of all 176 analyses. Most often for the comparison between Germany and Argentina.

Fig. S2d

*Note.* The figure displays the results from a series of Differential Item Functioning (DIF) analyses for item PFM33 using a multiverse approach. Each specification number on the horizontal axis represents a unique combination of analytical factors, including country comparisons, age corrections, parameter estimation methods, and DIF detection criteria. The purple colour indicates the presence of DIF, while the orange colour indicates the absence of DIF for each specification. Item PFM33 *Are you able to walk across a balance beam?* was flagged for DIF in 52.8% of all 176 analyses. Most often for the comparison between Germany and Argentina.

### Fig. S3. Test Information Function Plot


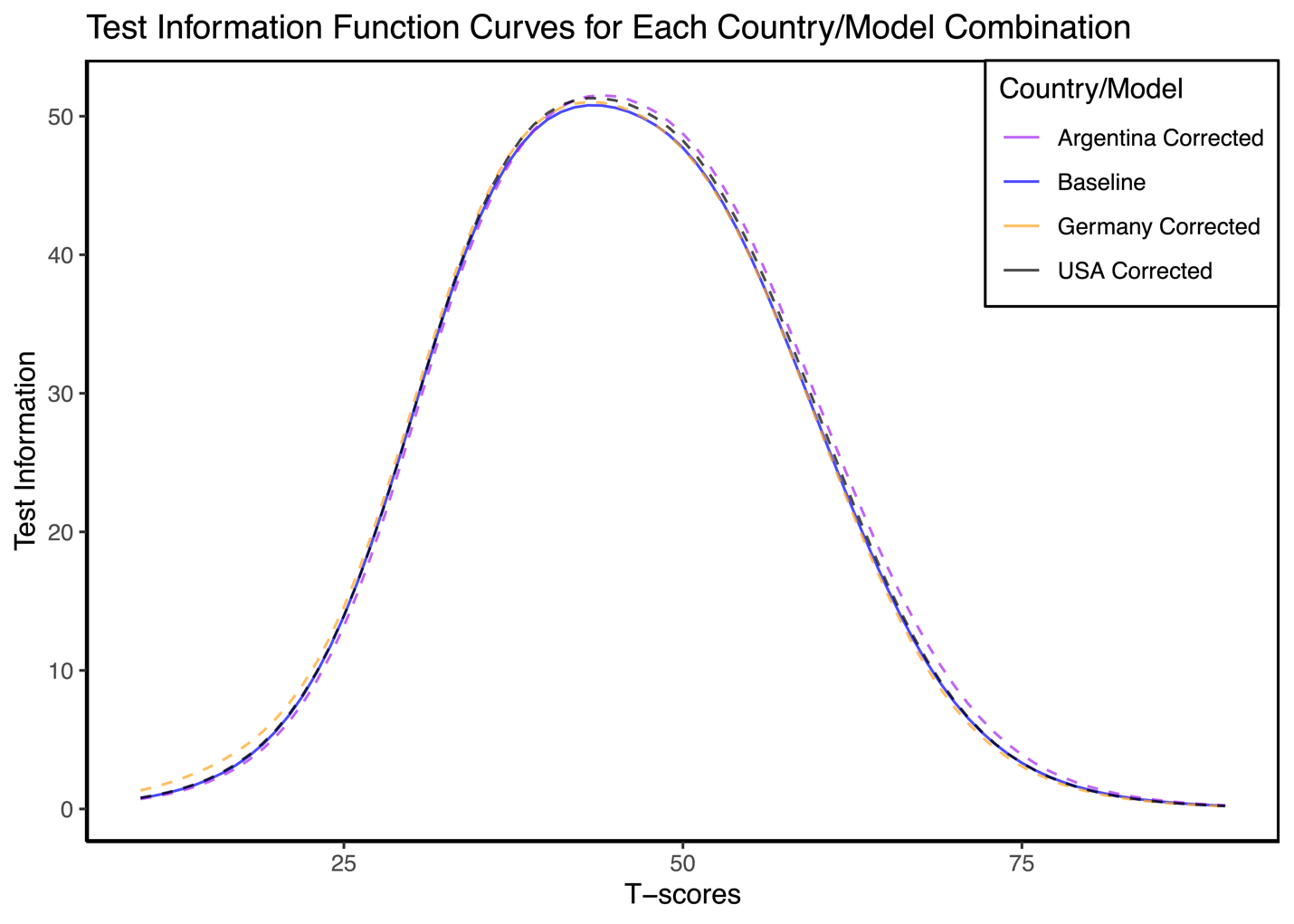


*Note. This graph shows test information function (TIF) curves for the Fully Invariant Model (baseline) with fixed item parameters across all countries (blue), and the models with freed parameters (corrected) for the identified DIF items (PFM 16, PFM 33, PFM 47, PFM 51) for each individual country (Argentina, Germany, USA). The close alignment of the curves indicates that the differences between the Fully Invariant and Partially Invariant Models are minimal.*

### Fig. S4 Test Characteristic Curve Plot


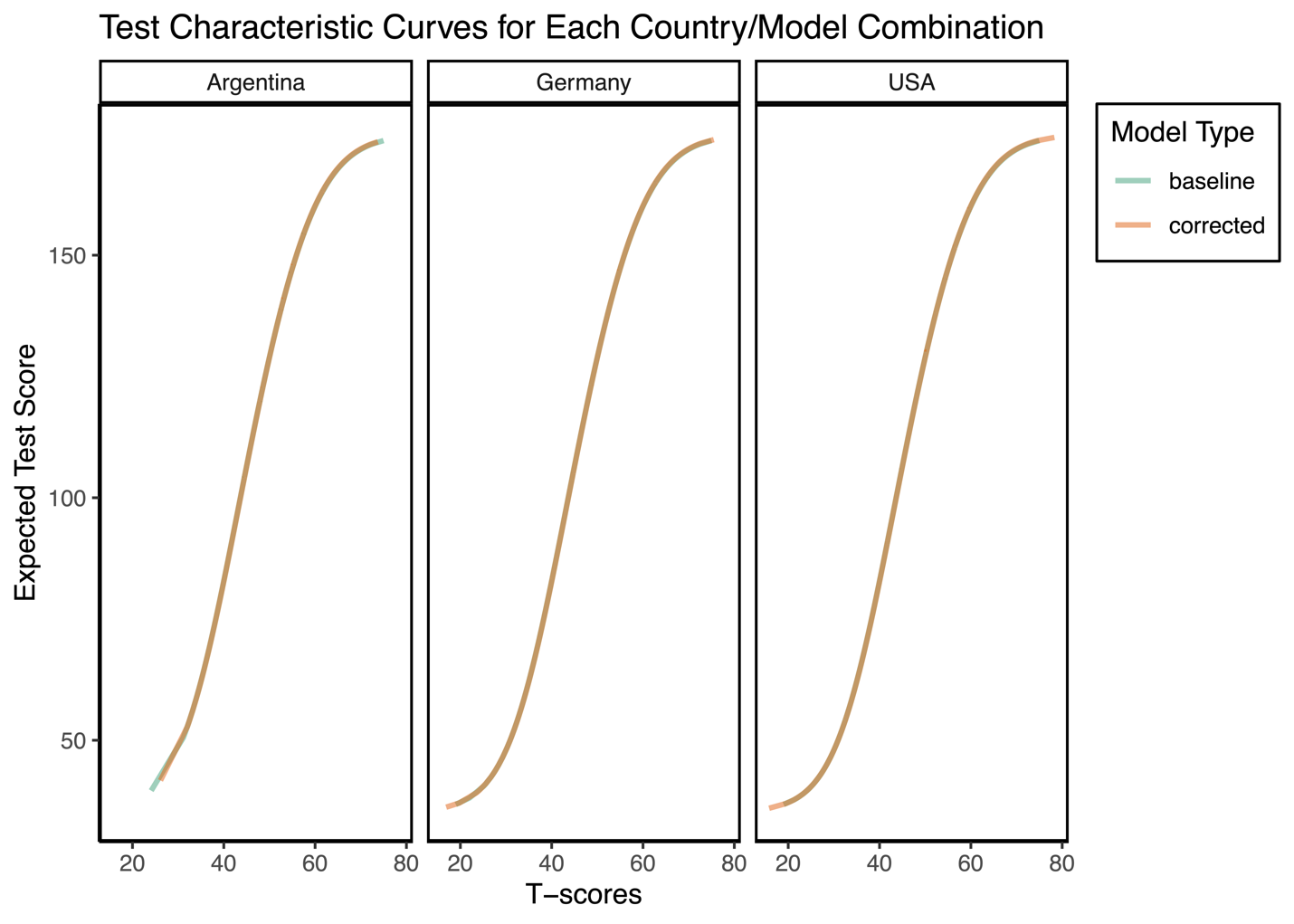


Note. This graph shows the test characteristic curves (TCC) comparing the baseline (fully invariant) model, where all item parameters are equal across countries, and the corrected (partially invariant) model, where the item parameters for identified DIF items are estimated freely for each country. For Argentina, Germany, and the USA, the stability observed in the TCC plots post-correction signals that the modifications made to address DIF did not result in significant alterations in how the test scores relate to the underlying latent trait. The close alignment of the curves indicates that the differences between the Fully Invariant and Partially Invariant Models are minimal.

### Fig. S5: Graphical display of the item PFM16 “Are you able to pass a 20-pound (10 kg) turkey or ham to other people at the table?” which shows uniform DIF with respect to country.

*Note.* The top-left graph presents the item characteristic curves (ICCs) for the item, contrasting the USA (black), Germany (orange dashed), and Argentina (purple dotted), the slope and category threshold values by country are printed on the graph. The top-right graph illustrates the absolute differences in ICCs between the USA and the other two countries, highlighting that the major disparities for the USA with both Germany and Argentina occur at lower levels of physical function (theta). The bottom-left graph depicts the item response functions for each group, derived from demographic-specific item parameter estimates (slope and category threshold values), which are also annotated on the graph. Finally, the bottom-right graph displays the weighted absolute difference in ICCs (referencing the top-right graph) based on the score distribution of the reference group, the USA, showing a negligible impact.

### Fig. S6: Graphical display of the item PFM33 “Are you able to walk across a balance beam?” which shows uniform DIF with respect to country.

*Note*. The upper-left graph illustrates the item characteristic curves (ICCs) for the item, comparing the USA (black), Germany (orange dashed), and Argentina (purple dotted), the slope and category threshold values by country are printed on the graph. The upper-right graph portrays the absolute differences in ICCs between the USA and the other two countries, showing that the primary differences are at lower levels with Germany and at medium levels with Argentina in terms of physical function (theta). The lower-left graph presents the item response functions for each group, based on demographic-specific item parameter estimates (slope and category threshold values), which are also indicated on the graph. Finally, the lower-right graph demonstrates the weighted absolute difference in ICCs (from the upper-right graph) according to the score distribution of the reference group, the USA, suggesting a minimal impact.

### Fig. S7: Graphical display of the item PFM46 “Are you able to pull a sled or a wagon with two children (total 100 lbs/50 kg) for 100 yards (100 m)?” which shows uniform DIF with respect to country.

*Note.* The top-left graph depicts the item characteristic curves (ICCs) for the item, showcasing comparisons between the USA (black), Germany (orange dashed), and Argentina (purple dotted), the slope and category threshold values by country are printed on the graph. The top-right graph details the absolute differences in ICCs for the USA against Germany and Argentina, highlighting that the primary differences for Argentina compared to the other two nations occur at medium levels of physical function (theta). The bottom-left graph presents the item response functions for each group, derived from demographic-specific item parameter estimates (slope and category threshold values), which are also noted on the graph. Finally, the bottom-right graph displays the weighted absolute difference in ICCs (referenced in the top-right graph) based on the score distribution of the reference group, the USA, demonstrating a negligible impact.

Fig. S8: Graphical display of the item PFM51 “Are you able to swim laps for 30 minutes at a moderate pace?” which shows non-uniform DIF with respect to country.

*Note.* The upper-left graph displays the item characteristic curves (ICCs) for the item, contrasting the USA (black) with Germany (orange dashed) and Argentina (purple dotted), the slope and category threshold values by country are printed on the graph. The upper-right graph demonstrates the absolute differences in ICCs for the USA compared to Germany and Argentina, revealing that the differences primarily occur at lower levels with Germany and at medium levels with Argentina in terms of physical function (theta). The lower-left graph presents the item response functions for each group, based on demographic-specific item parameter estimates (slope and category threshold values), also detailed on the graph. Finally, the lower-right graph illustrates the weighted absolute difference in ICCs (referenced in the upper-right graph) according to the score distribution of the reference group, the USA showing a minimal impact.

Table S1. Corrected item parameters for items flagged for differential item functioning.

| **Item** | **Country** | **a** | **b1** | **b2** | **b3** | **b4** |
| --- | --- | --- | --- | --- | --- | --- |
| PFM16 | USA | 2.09518 | -2.15984 | -1.49237 | -0.84221 | 0.043906 |
| PFM16 | Germany | 1.997836 | -3.21604 | -2.06141 | -1.20078 | -0.43299 |
| PFM16 | Argentina | 2.62817 | -1.64852 | -0.98199 | -0.32055 | 0.421307 |
| PFM33 | USA | 1.955181 | -1.16282 | -0.60458 | -0.02428 | 0.889324 |
| PFM33 | Germany | 2.202331 | -1.58368 | -1.01028 | -0.36081 | 0.531196 |
| PFM33 | Argentina | 1.901797 | -0.63697 | -0.13395 | 0.596486 | 1.456334 |
| PFM46 | USA | 2.469416 | -1.3926 | -0.81185 | -0.16817 | 0.68812 |
| PFM46 | Germany | 2.258541 | -1.46534 | -0.81943 | -0.19838 | 0.658638 |
| PFM46 | Argentina | 2.354495 | -0.67989 | -0.11818 | 0.614531 | 1.417107 |
| PFM51 | USA | 1.743609 | -0.77027 | -0.31615 | 0.360663 | 1.324552 |
| PFM51 | Germany | 1.536306 | -1.4493 | -0.79901 | -0.11058 | 0.806166 |
| PFM51 | Argentina | 1.594497 | -1.01923 | -0.36131 | 0.446386 | 1.496123 |
